# Diverse Partitiviruses From the Phytopathogenic Fungus, *Rosellinia necatrix*

**DOI:** 10.3389/fmicb.2020.01064

**Published:** 2020-06-26

**Authors:** Paul Telengech, Sakae Hisano, Cyrus Mugambi, Kiwamu Hyodo, Juan Manuel Arjona-López, Carlos José López-Herrera, Satoko Kanematsu, Hideki Kondo, Nobuhiro Suzuki

**Affiliations:** ^1^Institute of Plant Science and Resources, Okayama University, Kurashiki, Japan; ^2^Institute for Sustainable Agriculture, Spanish Research Council, Córdoba, Spain; ^3^Institute of Fruit Tree Science, National Agriculture and Food Research Organization (NARO), Morioka, Japan

**Keywords:** partitivirus, dsRNA virus, phytopathogenic fungus, *Rosellinia necatrix*, *Cryphonectria parasitica*, diversity, reassortment, horizontal transfer

## Abstract

Partitiviruses (dsRNA viruses, family *Partitiviridae*) are ubiquitously detected in plants and fungi. Although previous surveys suggested their omnipresence in the white root rot fungus, *Rosellinia necatrix*, only a few of them have been molecularly and biologically characterized thus far. We report the characterization of a total of 20 partitiviruses from 16 *R. necatrix* strains belonging to 15 new species, for which “*Rosellinia necatrix partitivirus 11*–*Rosellinia necatrix partitivirus 25*” were proposed, and 5 previously reported species. The newly identified partitiviruses have been taxonomically placed in two genera, *Alphapartitivirus*, and *Betapartitivirus*. Some partitiviruses were transfected into reference strains of the natural host, *R. necatrix*, and an experimental host, *Cryphonectria parasitica*, using purified virions. A comparative analysis of resultant transfectants revealed interesting differences and similarities between the RNA accumulation and symptom induction patterns of *R. necatrix* and *C. parasitica*. Other interesting findings include the identification of a probable reassortment event and a quintuple partitivirus infection of a single fungal strain. These combined results provide a foundation for further studies aimed at elucidating mechanisms that underly the differences observed.

## Introduction

Plant pathogenic fungi provide platforms for identifying eukaryotic viruses (Pearson et al., 2009; Xie and Jiang, 2014; Ghabrial et al., 2015; Suzuki, 2017; Hillman et al., 2018). Research performed in previous decades have revealed that the diversity of fungal viruses or mycoviruses is much greater than previously imagined (Yu et al., 2010; Kondo et al., 2013a; Liu et al., 2014; Kanhayuwa et al., 2015; Zhang et al., 2016). Further, studies involving particular fungal-viral systems have provided interesting insights into virus-virus and virus-host interactions (Cho et al., 2013; Jiang et al., 2013; Xie and Jiang, 2014; Hillman et al., 2018). Antagonistic, mutualistic and neutral virus/virus interactions, and beneficial, harmful, or no effects on their hosts have been reported. Among fungal hosts commonly used for the study of viruses is the phytopathogenic ascomycete fungus, *Rosellinia necatrix*, which infects over 400 plant species and causes white root rot in perennial crops worldwide, and particularly in Japan (Pliego et al., 2012; Kondo et al., 2013b). A survey of over 1,000 field isolates showed that ~20% of field isolates harbor diverse populations of viruses (Arakawa et al., 2002; Ikeda et al., 2004; Kondo et al., 2013b; Zhang et al., 2014, 2016; Arjona-Lopez et al., 2018). Importantly, the characterization of some viruses has led to the approval of proposals to create new viral families by the International Committee on Taxonomy of Viruses (ICTV) (Chiba et al., 2009; Lin et al., 2012). The majority of viruses detected in *R. necatrix* have been partitiviruses (Yaegashi et al., 2013) (Kanematsu and Sasaki, unpublished results), which was also the case for the fungal forest inhabitant, *Heterobasidion* spp. (Vainio and Hantula, 2016).

Partitiviruses are bisegmented, biparticulate viruses with double-stranded RNA (dsRNA) genomes that range from 3.0 to 4.8 kbp (Nibert et al., 2014; Vainio et al., 2018a). With a few exceptions, RNA-dependent RNA polymerase (RdRP) is encoded within the large segment, while capsid protein (CP) is encoded within the smaller segment. Partitiviruses typically form icosahedral particles that are 30–35 nm in diameter. The near-atomic resolution of ~5 Å has been achieved for the *T* = 1 capsids of partitiviruses via cryo-electron microscopy (Nibert et al., 2013). Based on phylogenetic relationships and host ranges, partitiviruses are now classified into 5 genera: *Alphapartitivirus, Betapartitivirus, Gammapartitivirus, Deltapartitivirus*, and *Cryspovirus* (Nibert et al., 2014; Vainio et al., 2018a). The first two genera contain members that infect both plant and fungal species, while the genera *Gammapartitivirus* and *Deltapartitivirus* are composed of members that infect exclusively fungal and plant species, respectively. In this regard, it should be noted that fungus-infecting, betapartitivirus-related sequences are detected in various plant genomes, a paleological record of their capacity to infect plant species (viral fossils or endogenous virus elements) and their association with plants several tens of millions of years ago (Liu et al., 2010; Chiba et al., 2011). Cryspoviruses have been isolated from protozoa of the genus *Cryptosporidium* that infect mammals as a parasite. Recently, the novel genera “*Epsilonpartitivirus*” and “*Zetapartitivirus*” have been proposed for some novel viruses or virus-like agents associated with fungi and arthropods (Nerva et al., 2017a; Jiang et al., 2019). Partitiviruses, in most cases, produce symptomless infections. Exceptions include growth-reducing Heterobasidion partitivirus 3 (an alphapartitivirus) (Vainio et al., 2010) and Aspergillus fumigatus partitivirus 1 (a gammapartitivirus) (Bhatti et al., 2011), and hypovirulence-conferring partitiviruses such as *Rhizoctonia solani* partitivirus 2 (an alphapartitivirus, RsPV2) (Zheng et al., 2014), Sclerotinia sclerotiorum partitivirus 1 (a betapartitivirus, SsPV1) (Xiao et al., 2014), Aspergillus flavus partitivirus 1 (a zetapartitivirus) (Jiang et al., 2019) and Heterobasidion partitivirus 13 (an alphapartitivirus) (Vainio et al., 2018b). In other cases, some partitiviruses have been shown to contribute to hypovirulence and/or growth defects induced by other co-infecting fungal dsRNA viruses such as megabirnavirus and chrysovirus (Wang et al., 2014; Sasaki et al., 2016).

Despite the large number of partitiviruses identified thus far (Nibert et al., 2014; Vainio et al., 2018a), only a few including *R. necatrix-*infecting partitiviruses (Rosellinia necatrix partitiviruses: RnPV1, RnPV2, RnPV3, RnPV6, RnPV7, RnPV8, RnPV9, and RnPV10) have been identified (Sasaki et al., 2006, 2007, 2016; Chiba et al., 2013a, 2016; Yaegashi and Kanematsu, 2016; Arjona-Lopez et al., 2018). Biological characterization of fungal viruses has been facilitated by the development of improved virion transfection methods (Hillman et al., 2004). Fungal partitiviruses, unlike plant partitiviruses, can be experimentally introduced into both original and experimental hosts. The method has previously been used to study some partitiviruses including RnPV1, RnPV2, RnPV6, SsPV1, and RsPV2 (Sasaki et al., 2007; Chiba et al., 2013a, 2016; Xiao et al., 2014; Zheng et al., 2014). In this regard, it is important to note that the phytopathogenic ascomycete that causes chestnut blight, *Cryphonectria parasitica*, is a model filamentous host used to explore virus-virus and virus-host interactions. The fungus is an attractive host because it is biologically tractable, genetically manipulatable, and can support many homologous and heterologous fungal viruses (Eusebio-Cope et al., 2015). In addition, a number of biological resources and molecular tools are available for the fungus (Nuss, 2005; Eusebio-Cope et al., 2015; Chiba et al., 2018). Therefore, the use of *C. parasitica* as a viral host has facilitated the study of viral symptom induction via host antiviral RNA silencing to and counter defense of both homologous and heterologous fungal viruses (Faruk et al., 2008; Andika et al., 2017, 2019). Recent advancements made by using this fungus as a model system include the characterization of mechanisms of induction of and susceptibility to mechanisms of antiviral RNA silencing of different fungal viruses (Chiba and Suzuki, 2015; Andika et al., 2017, 2019). It has also been shown that a fungal gammapartitivirus replicates in plant cells (Nerva et al., 2017b).

Here, we report the characterization of a total of 20 partitiviruses belonging to 15 new and 5 previously-identified species placed in the genus *Alphapartitivirus* or *Betapartitivirus*. Some tested partitiviruses differ with respect to viral content in both *R. necatrix* and *C. parasitica*, and symptom induction in the experimental host. This study has enhanced our understanding of the molecular and biological diversity of fungal partitiviruses.

## Materials and Methods

### Fungal and Viral Materials

A total of 16 field isolates of *R. necatrix* listed in Table 1 were used in the study. Most fungal isolates were collected from Japanese pear or apple trees in Saga, Fukuoka, Hyogo, Nagano, and Gunma Prefectures, Japan, while Rn459 was collected from an avocado tree in Malaga, Spain (Arakawa et al., 2002; Ikeda et al., 2004; Yaegashi et al., 2013; Arjona-Lopez et al., 2018). The Japanese fungal strains had earlier been shown to harbor partiti-like dsRNAs of ~2.0–2.5 kbp by a conventional dsRNA extraction method (Arakawa et al., 2002; Ikeda et al., 2004; Sasaki and Kanematsu, unpublished data). The partitivirus isolate from the Spanish strain, Rn459, was previously partially sequenced (Arjona-Lopez et al., 2018). The standard strain, W97, of *R. necatrix* has been described in previous reports (Kanematsu et al., 2010; Shimizu et al., 2018). The standard *C. parasitica* strain, EP155, and its *dicer-like 2* (*dcl2*) knockout (KO) mutant strain, Δ*dcl2*, were a generous gift from Dr. Donald L. Nuss (University of Maryland, College Park, MD). The Δ*dcl2* strain carries deletion of a key gene used for antiviral RNA silencing, *dcl2* (Segers et al., 2007). All fungal materials were cultured either on potato dextrose agar (PDA, BD Difco Laboratories, Detroit, MI, USA) plates or potato dextrose broth (PDB, BD Difco Laboratories).

**Table 1:** List of *Rosellinia necatrix* isolates used.

**Isolate^a^**	**Collection locality**	**Host plants**	**Collection year**	**MCG^b^**	**Group of NGS**	**Virus detected^e^**	**Source**
W98	Saga	Japanese pear	1998	80	Pool-3/2	RnPV11	Ikeda et al., 2004
W118	Saga	Japanese pear	1998	86	Pool-3/1	RnPV3, 12, 13	Ikeda et al., 2004
W129	Fukuoka	Japanese pear	1998	88	Pool-2	RnPV25	Ikeda et al., 2004
W442	Hyogo	Unknown	2000	169	Pool-3/1	RnPV18, 19	This study
W558	Saga	Japanese pear	1998	85	Pool-1	RnPV6	Ikeda et al., 2004
W662	Gunma	Apple	2000	301	Pool-1	RnPV23, 24	Ikeda et al., 2004
W744	Saga	Japanese pear	2001	325	Pool-3/2	RnPV1, 14–17	Ikeda et al., 2004
W1030	Nagano	Apple	2009	139	NA^c^	RnPV4	Yaegashi et al., 2013
W1031	Nagano	Apple	2009	139	Pool-4	RnPV3, 4	Yaegashi et al., 2013
W1040	Nagano	Apple	2009	139	Pool-1	RnPV5 (+one)^f^	Yaegashi et al., 2013
W1041	Nagano	Apple	2009	139	NA^c^	RnPV5	Yaegashi et al., 2013
W1050	Nagano	Apple	2009	139	Pool-4	RnPV22	Yaegashi et al., 2013
W1126	Iwate	Lacquer tree	2011	U1	NA^c^	RnPV22	From Dr. M. Tabata
W1134	Kagawa	Lacquer tree	2011	U13	Pool-3/1	RnPV20, 21	From Dr. M. Tabata
W1135	Kagawa	Lacquer tree	2011	U14	NA^c^	RnPV20	From Dr. M. Tabata
Rn459	Malaga	Avocado	2016	unknown	NA^d^	RnPV10	Arjona-Lopez et al., 2018

a *All isolates were collected in Japan, except for Rn459 isolated from Spain*.

b *Mycelial compatibility group*.

c *Not applicable. For these viruses, conventional cDNA libraries were constructed and subjected to Sanger sequencing*.

d *Partial genomic sequence was reported by Arjona-Lopez et al. (2018)*.

e *Viral infection was confirmed by RT-PCR*.

f *The partial genome sequence derived from an additional potential novel partitivirus besides RnPV5 was detected in the W1040 strain*.

### Partitivirus Purification, Electron Microscopy, and Transfection

Partitivirus particles were semi-purified following the method described by Chiba et al. (2016). After differential centrifugation, viral fractions were subjected to sucrose/cesium chloride gradient ultracentrifugation. Purified virus preparations were examined by transmission electron microscopic (TEM, H-7650 Hitachi, Tokyo, Japan) observation after EM staining (the EM stainer, an alternative for uranyl acetate, Nissin EM Co., Tokyo, Japan) (Nakakoshi et al., 2011). Virus preparations were loaded on sodium dodecyl sulfate (SDS)—polyacrylamide (10%) gel and electrophoreses, and then stained with Rapid stain CBB kit (Nacalai tesque inc., Kyoto Japan). Semi-purified particle fractions were used to transfect virus-free protoplasts derived from either strain W97 from *R. necatrix*, or EP155 and Δ*dcl2* strains from *C. parasitica*. Transfection of strain W97 was conducted according to the method described by Kanematsu et al. (2010), and transfection of *C. parasitica*-derived strains was performed according to the method described by Salaipeth et al. (2014). After regeneration, partitiviral infection of candidate transfectants were assessed using the one-tube RT-PCR method using PrimeScript™ One Step RT-PCR Kit ver.2 (Takara Bio Inc., Shiga Japan) and toothpicks described by Urayama et al. (2015).

### RNA Analyses

Viral genomic dsRNA sequences were determined using two approaches: conventional complimentary DNA (cDNA) library construction using purified viral dsRNA and subsequent Sanger sequencing (Chiba et al., 2016), and next-generation high-throughput sequencing (NGS) of total RNA from fractions obtained from infected mycelia (Shamsi et al., 2019). dsRNA was isolated from mycelia cultured in PDB media for 1 week as previously described (Chiba et al., 2013a). Total RNA, dsRNA or single-stranded RNA (ssRNA) fractions were obtained from *R. necatrix* strains as described by Chiba et al. (2013a).

For RNA-Seq analyses, RNA samples were pooled into four groups: two were comprised of dsRNA samples and were named Pool-1 and -2 (1.2 and 0.9 μg, respectively) and other two, Pool-3 and -4, contained total RNA samples (14.1 and 67.5 μg, respectively) (see Table 1). Each RNA pool with/without ribosomal RNA depletion treatment (the Ribo-Zero kit, Illumina, San Diego, CA, USA) was subjected to cDNA library construction (the TruSeq RNA Sample Preparation kit v2, Illumina) and pair-end deep sequencing (100 bp pair-end reads) using the Illumina HiSeq 2500/4000 platforms (Illumina) performed by Macrogen Inc. (Tokyo, Japan). After deep sequencing, adapter-trimmed sequence reads (Pool-1: 28,827,612; Pool-2: 29,248,296, Pool-3: 46,288,894, and Pool-4: 47,084,068 raw reads) were *de novo* assembled using the CLC Genomics Workbench (version 11, CLC Bio-Qiagen, Aarhus, Demark). To verify the virus infection in the fungal strains, we performed RT-PCR using the specific primer sets for each of the partitivirus (Supplementary Table S1). Viral genomic sequences were completed by RLM-RACE (RNA ligase mediated rapid amplification of complementary DNA ends) and gap-filling RT-PCR (Suzuki et al., 2004; Chiba et al., 2009) (Supplementary Table S1). Sequences obtained in this study were deposited in EMBL/GenBank/DDBJ databases with accession numbers LC517370–LC517399, as described in Table 2.

**Table 2:** Properties of newly discovered partitiviruses from *Rosellinia necatrix*.

**Virus name^a^**	**Original host strain**	**Contig No**.	**Total read count**	**Genus**	**Segment length^b^**	**Blastp**		**Protein size**	**Symptom^c^**		
					**dsRNA1/(accession)**	**Hit with highest score in**	**Identity**	**RdRp (aa)**	***R.n*.**	***C.p***.	
					**dsRNA2 (accession)**	**Blastp**	**(%)**	**Cp (aa)**		**Δ*dcl2***	**EP155**
RnPV11	W98	4 262	61129 26713	*Beta-*	2445 (LC517370) 2326 (LC517371)	Rosellinia necatrix partitivirus 20 Fusarium poae partitivirus 2	71 35	757 652	–~+	++	+++
RnPV12	W118	148 779	47804 1197	*Alpha-*	1845 LC517372 1925 (LC517373)	Botrytis cinerea partitivirus 2 Erysiphe necator partitivirus 1	63 38	551 533	–~+		
RnPV13	W118	610 529	6211 4558	*Alpha-*	1965 (LC517374)^*^ 1822 (LC517375)	Rhizoctonia oryzae-sativae partitivirus 1 Rhizoctonia oryzae-sativae partitivirus 1	59 54	469 488			
RnPV3 ^f^	W118	317 347		*Beta-*	2246 (LC010950) 2065 (LC010951)	Rosellinia necatrix partitivirus 3 Rosellinia necatrix partitivirus 3	99 96	709 613		–	–
RnPV14	W744	118 750	46844 2477	*Beta-*	2412 (LC517376) 2427 (LC517377)	Rosellinia necatrix partitivirus 4 Rosellinia necatrix partitivirus 1-W8	72 47	743 706	–~+	++	
RnPV15	W744	826 2267	7917 3491	*Beta-*	2517 (LC517378) 2358 (LC517379)	Rosellinia necatrix partitivirus 14 Rosellinia necatrix partitivirus 16	59 49	747 663			
RnPV16	W744	509 3216	20228 944	*Beta-*	2372 (LC517380) 2344 (LC517381)	Podosphaera prunicola partitivirus 4 Rosellinia necatrix partitivirus 15	61 49	730 662		++	+++
RnPV17	W744	671 237	4185 8985	*Beta-*	2292 (LC517382) 2235 (LC517383)	Heterobasidion partitivirus 7 Rhizoctonia solani partitivirus 7	63 71	725 670			
RnPV1^f^	W744/ W1134	43 82/69		*Beta-*	2374 (AB113347) 2263 (AB113348)	Rosellinia necatrix partitivirus 1-W8 Rosellinia necatrix partitivirus 1-W8	98 99/94	709 686			
RnPV18^d^	W442	110 874	30695	*Beta-*	2410 (LC517384) 2337 (LC517385)	Enteoleuca partitivirus 2 Dill cryptic virus 2	99.7 43	740 670	–	–	–
RnPV19^d^	W442	26 379	40899 5244	*Alpha-*	2013 (LC517386) 1842 (LC517387)	Entoleuca partitivirus 1 Grosmannia clavigera partitivirus 1	99.7 42	606 520		+	–
RnPV20	W1134	5 7	186156 55670	*Beta-*	2417 (LC517388) 2318 (LC517389)	Rosellinia necatrix partitivirus 11 Ceratobasidium partitivirus CP-h	71 61	755 643	–~+	+++	+++
RnPV21	W1134	161 69	49075 3784	*Beta-*	2352 (LC517390) 2361 (LC517391)	Podosphaera prunicola partitivirus 4 Rosellinia necatrix partitivirus 1-W8	65 94	721 686			
RnPV22	W1050/ W1126	27 707		*Alpha-*	2012 (LC517392) 2037 (LC517393)	Trichoderma atroviride partitivirus 1 Trichoderma atroviride partitivirus 1	76 43	613 584			
RnPV23	W662	127 25		*Alpha-*	1831 (LC517394)^**^ 1791 (LC517395)^**^	Heterobasidion partitivirus 13 Heterobasidion partitivirus 13	49 37	571 514			
RnPV24	W662	77 125		*Alpha-*	1946 (LC517396)^**^ 1771 (LC517397)^**^	Oyster mushroom isometric virus II Medicago sativa alphapartitivirus 1	75 28	598 498			
RnPV25	W129	51 188		*Beta-*	2374 (LC517398)^**^ 2049 (LC517399)^**^	Trichoderma citrinoviride partitivirus 1 Trichoderma citrinoviride partitivirus 1	65 54	733 642			
RnPV10	Rn459			*Alpha-*	1896 (LC333736) 1911 (LC333737)	Rosellinia necatrix partitivirus 10 Rosellinia necatrix partitivirus 10	100 100	573 539			
RnPV4^e^	W1030/ W1031	4 270		*Alpha-*	2342 (AB698493) 2295 (LC521312)	Rosellinia necatrix partitivirus 4 Sclerotinia sclerotiorum partitivirus 1	100 48	744 683			
RnPV5^e^	W1040/ W1041	10/522 27/62		*Beta-*	2046 (AB698494) 1906 (LC521313)	Rosellinia necatrix partitivirus 5 Trichoderma atroviride partitivirus 1	99.7 44	647 576			
RnPV6^f^	W558	52 137		*Beta-*	2499 (LC010952) 2462 (LC010953)	Rosellinia necatrix partitivirus 6 Rosellinia necatrix partitivirus 6	100 100	756 729	–~+	+++	+++

a *RnPV22 was also detected from the W1126 strain*.

b* *5′-terminal sequence of dsRNA1 is incomplete*;

** *5′- and 3′-terminal sequences of both segments are incomplete. Underlined: RdRp is encoded by the smaller dsRNA segments*.

c *R.n., R. necatrix W97 strain; C.p., C. parasitica EP155 or* Δ*dcl2 strain. Growth reduction: –, No; +: Slight; ++, Moderate; +++, Great. See Figure 4 and Supplementary Figure S4 for colony morphology of virus-infected fungal strains*.

d,e *Near-complete or complete dsRNA1 sequences of these viruses from Spanish Entoleuca sp. (d) or R. necatrix (e) have already been deposited in GenBank*.

f *Partitiviruses belonging to the same species have previously been reported from different Japanese strains of R. necatrix*.

### Bioinformatics

Viral sequences were analyzed with online bioinformatics tools as described by Kondo et al. (2015). After *de novo* assembly, contigs [Pool-1: 23,341 (~9.9 kb), Pool-2: 23,130 (~9.8 kb), Pool-3: 10,485 (~13.2 kb), and Pool-4: 11,016 (~13.8 kb)] were subjected to local BLAST searches against the viral reference sequence (RefSeq) dataset from the National Center for Biotechnology Information (NCBI). Viral-like sequences were analyzed using Enzyme X v3.3.3^1^ or GENETYX-MAC (Genetyx Co., Tokyo, Japan). Database searches of viral sequences were performed using the BLAST (BLASTn and BLASTp) programs available from NCBI. Pairwise amino acid identity was calculated using SDT v1.2 (Muhire et al., 2014).

Phylogenetic reconstruction was carried out using the maximum-likelihood (ML) method as described previously (Kondo et al., 2019). Deduced amino acid sequences from virus RNA sequences were aligned using MAFFT version 7 (Katoh and Standley, 2013) and unreliably aligned regions were eliminated using Gblocks 0.91b (Talavera and Castresana, 2007). ML phylogenetic trees were constructed by PhyML 3.0 (Guindon et al., 2010) using automatic model selection via smart model selection (SMS) (Lefort et al., 2017). Support for branches was examined via bootstrapping with 1,000 repetitions. The phylogenetic trees (mid-point rooted) were visualized and refined using FigTree version 1.3.1 software^2^.

## Results

### dsRNA Profiles of *R. necatrix* Isolates

**Figure 1:**
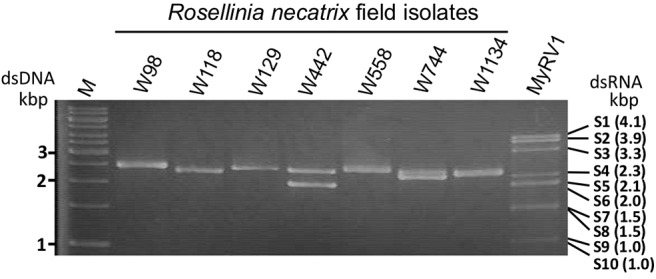
Agarose gel electrophoresis of dsRNA isolated from field isolates of *Rosellinia necatrix*. dsRNA fractions were obtained from fungal isolates as indicated above each lane, and electrophoresed through a 1% agarose gel in 1 × TAE (40 mM Tris-acetate−1 mM EDTA, pH 7.8). See Table 1 for information regarding fungal isolates. A 1-kb DNA ladder (Thermo Fisher Scientific., Inc., Waltham, MA, USA) and MyRV1 genomic dsRNA (Supyani et al., 2007) were used as size markers.

Field-collected strains of *R. necatrix* have previously been screened for mycoviruses by Japanese research groups (Arakawa et al., 2002; Ikeda et al., 2004; Yaegashi and Kanematsu, 2016). We selected a total of 16 fungal strains harboring ~1.5–2.5 kbp dsRNAs for the study (Table 1). dsRNA profiles of some these strains have been reported previously by Ikeda et al. (2004), Yaegashi et al. (2013), and Arjona-Lopez et al. (2018). Bands were confirmed to be dsRNA following double digestion using RQ1 RNase free DNase I (Promega) and S1 Nuclease (Takara) enzymes (Eusebio-Cope and Suzuki, 2015). dsRNA-enriched or total RNA fractions and their mixed pools (Poo1-1–4) were then subjected to either conventional Sanger sequencing of cDNA clones or NGS analyses, respectively (Table 2; see Figure 1 for the dsRNA profile of Pool-3 from *R. necatrix-*derived strains). It is worth noting that some dsRNA bands of the sequence analysis turned out to be doublets (or more).

### Sequence Analysis

Properties of fully and near-fully sequenced partitiviruses are summarized in Table 2. Some partially characterized partitiviruses, i.e., RnPV1, RnPV3, RnPV4, RnPV5, RnPV6, and RnPV10, were previously isolated from *R. necatrix* strains (Sasaki et al., 2005; Chiba et al., 2013b, 2016; Yaegashi et al., 2013; Arjona-Lopez et al., 2018). The partial genomic sequences of RnPV4, RnPV5, and RnPV10, and the complete genomic sequences of RnPV1 and RnPV6 were previously determined. The remainder of the partitivirus isolates for which novel sequence information was obtained in this study were designated Rosellinia necatrix partitivirus 11–25 (RnPV11–RnPV25) (Table 2).

NGS and conventional sequencing, followed by subsequent RT-PCR analyses, suggested that tested fungal strains were either singly (W98, W129, W558, W1030, W1041, W1050, W1126, W1135, and Rn459), doubly (W442, W662, W1031, W1040, and W1134), triply (W118) or quintuply (W744) infected by partitiviruses (Tables 1, 2). Partitivirus infection was confirmed by RT-PCR using specific primer sets designed from NGS contigs or sequences by conventional methods (Table 2 and Supplementary Table S1). Particularly, it is noteworthy that W744 was infected by five distinct partitiviruses, RnPV1, RnPV14, RnPV15, RnPV16, and RnPV17, as has also been reported in some other fungal hosts multiply infected by mitoviruses (Hillman et al., 2018). When the nucleotide sequences of these co-infecting viruses were compared, the highest sequence identity is 59% detected between RnPV14 dsRNA1 and RnPV15 dsRNA1 (see Supplementary Figure S3 for amino acid sequence comparison), which is low enough to avoid possible RNA silencing-mediated inter-virus antagonism. Fifteen novel partitiviruses, including those identified by conventional sequencing methods, from seven fungal strains such as W98 (RnPV11), W118 (RnPV12 and 13), W442 (RnPV18 and 19), W744 (RnPV14–17), W1050/1126 (dsRNA segments with conventional sequencing, RnPV22), W1134 (RnPV20 and 21), and Rn459 (two reported dsRNA contigs for RnPV10, Arjona-Lopez et al., 2018) had two segments that were completely sequenced using the RLM-RACE method (Table 2). In addition, the only fully-sequenced coding regions of the three other novel partitiviruses were from the fungal strains W662 (RnPV23 and 24) and W129 (RnPV25) (Table 2).

Sequence analysis revealed that each segment encoded one open reading frame (ORF), and BlastP analysis confirmed that the ORF from dsRNA1 (larger dsRNA segments, in general) encoded RdRP, which was closely related to alpha- or betapartitiviruses of the *Partitiviridae* family (Table 2, see below). The ORF from dsRNA2 (smaller dsRNA segments, in general) encoded CP, which was also related to other known alpha- or betapartitiviruses. Viral sequences from *R. necatrix* from W744 (RnPV1), W118/1031 (RnPV3), W1030/1031 (RnPV4, dsRNA1 segment determined by conventional sequencing), W1041/1040 (RnPV5) and W558 (RnPV6) strains were 85–100% identical to known *R. necatrix* partitiviruses RnPV1/W8, RnPV3/W1029, RnPV4/W1028 (dsRNA1), RnPV5/W1028 (dsRNA1), and RnPV6/W113, respectively (Table 2). For RnPV4 and RnPV5, previously undescribed dsRNA2 segments were newly sequenced (Table 2). Intriguingly, two dsRNA1 sequence for RnPV18 and 19, respectively, from the single W442 strain were 99% identical to two reported partitivirus sequences (Entoleuca partitivirus 2 and 1, EnPV2 and 1), respectively, which were both obtained from a Spanish fungal isolate (E97-14) of *Entoleuca* sp. entirely different from, but sympatric to, *R. necatrix* that infest avocado orchards in Spain (Velasco et al., 2019, 2020) (Table 2). Note that the dsRNA2 sequences of EnPV1 and 2 from the GenBank/EMBL/DDBJ databases were unavailable. The 5′-terminus of EnPV1 remains incomplete. Thus, we report the full-length sequence of the genome of RnPV18 and RnPV19, respectively (Table 2).

### Genome Organization, Protein Sequence Similarities, and Phylogenetic Analysis

**Figure 2:**
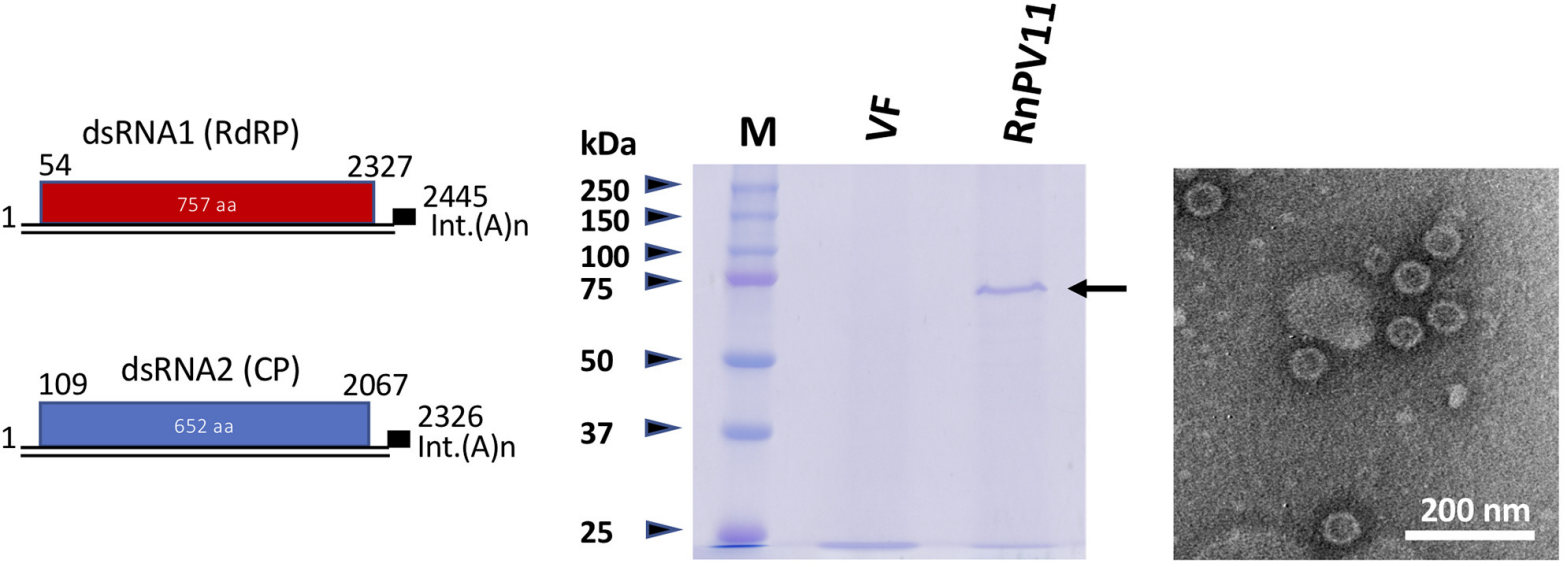
Schematic of the genome organization, particle protein composition, and morphology of a newly discovered partitivirus of *R. necatrix*. **(Left)** Genome organization of a representative betapartitivirus, RnPV11/W98. Sequence information for RnPV11 is summarized in Table 2. **(Middle)** SDS-PAGE analysis of purified preparations of RnPV11. Virus particles were purified from the mycelia of the W98 strain (lane RnPV11) and analyzed in 10% polyacrylamide gel electrophoresis. The arrow indicates the migration of RnPV11/W98 CP. A preparation was also obtained in parallel from virus-free W97 (VF). Protein size standards used (lane M, Precision Plus Protein Dual Color Standards) were purchased from Bio-Rad Laboratories, Inc., Hercules, CA, USA. **(Right)** Electron microscopy of RnPV11 virions. A purified virus preparation of RnPV11 was examined using a Hitachi electron microscope model H-7650.

All novel partitivirus strains from *R. necatrix* characterized molecularly have been determined to belong to either the genus *Alphapartitivirus* or *Betapartitivirus* (Table 2). No gammapartitiviruses, which exclusively infect fungi, were detected within *R. necatrix* as a part of this or previous studies (Sasaki et al., 2005; Chiba et al., 2013a; Yaegashi et al., 2013; Yaegashi and Kanematsu, 2016; Arjona-Lopez et al., 2018). Their sequence characteristics are similar to those of previously reported partitivirus members (Nibert et al., 2014). Namely, the size ranges of their segments, 5′- and 3′-untranslated regions (UTRs), and encoded proteins of these viruses mostly fall within ranges reported previously, whereas a few expand the reported size distributions within alpha- and betapartitiviruses (Nibert et al., 2014). The genome sizes of most reported alphapartitiviruses range from 3.6 to 3.9 kbp (Nibert et al., 2014), but RnPV22 isolate extended the range from 3.6 to 4.0 kbp. Similarly, an expansion of genome segment and coding protein size is also observed for betapartitiviruses RnPV6, RnPV14, and RnPV15. dsRNA1 segments of partitiviruses are usually longer than dsRNA2 segments, but we encountered few exceptions in RnPV12, RnPV14, RnPV21, and RnPV22, which had longer dsRNA2 than dsRNA1 segments (Table 2), as also has been reported for RnPV6 (Chiba et al., 2016). As an example, the genome organization, protein component, and virion morphology of a novel *R. necatrix* virus betapartitivirus, RnPV11, isolated from the strain W98, is shown in Figure 2. The RnPV11 genomic segments of ~2.4 and 2.3 kbp were shown to be encased in spherical particles of ~30 nm in diameter composed of the CP of ~72 kDa.

Partitiviruses analyzed in this study had terminal sequences similar to those described previously (Nibert et al., 2014; Vainio et al., 2018a). For several partitiviruses from *R. necatrix* isolates, such as RnPV19, RnPV20, and RnPV22, “CAA” repeats were present at the 5′-UTR region, which has been assumed to be a translation enhancer (Jiang and Ghabrial, 2004) (Supplementary Figure S1). The 20–30 nucleotides located at the 5′-terminal regions are highly conserved between genome segments, and the 5′-terminal-most nucleotides are considered consensus sequences and are shown in bold in Supplementary Table S2. The 5′-terminal G residue is strictly conserved at the terminal end or +1 from the end (Supplementary Figure S2). For 3′-termini, the plus strands of the genome segments of some betapartitiviruses, such as RnPV11, RnPV14, and RnPV15, end with interrupted poly(A) tracts, while those of other partitiviruses contain additional nucleotides, which include the di- or tri-nucleotide “UC,” “CC,” or “CU” that follow interrupted poly(A) tracts (Supplementary Table S2).

The BlastP (or BlastN as mentioned above) results are summarized in Table 2. The species demarcation criteria set by the ICTV are ≤ 90% and ≤ 80% amino acid identities for RdRP and CP, respectively (Vainio et al., 2018a). Of 20 newly sequenced alpha- or betapartitivirus isolates, 15 (including EnPV dsRNA segments) showed much smaller amino acid identities with known partitiviruses, which ranged 28–76% for RdRP or CP, than the above-mentioned species demarcation criteria (Table 2). In addition, pairwise comparisons of proteins encoded by new partitiviruses revealed moderate levels of amino acid sequence identity (less than above criteria) among RdRPs and CPs (Supplementary Figure S3). These results indicated that the 15 partitiviruses that were newly identified belong to new species, which were named Rosellinia necatrix partitivirus 11 to Rosellinia necatrix partitivirus 25. Notably, the nucleotide sequence identities of dsRNA1 and dsRNA2 of RnPV21/W1134 were most similar to Podosphaera prunicola partitivirus 4 (45%) and RnPV1/W8 or W744 (94%) (Table 2), respectively. This strongly suggests that a reassortment event likely occurred between RnPV1 and another partitivirus (see Discussion).

**Figure 3:**
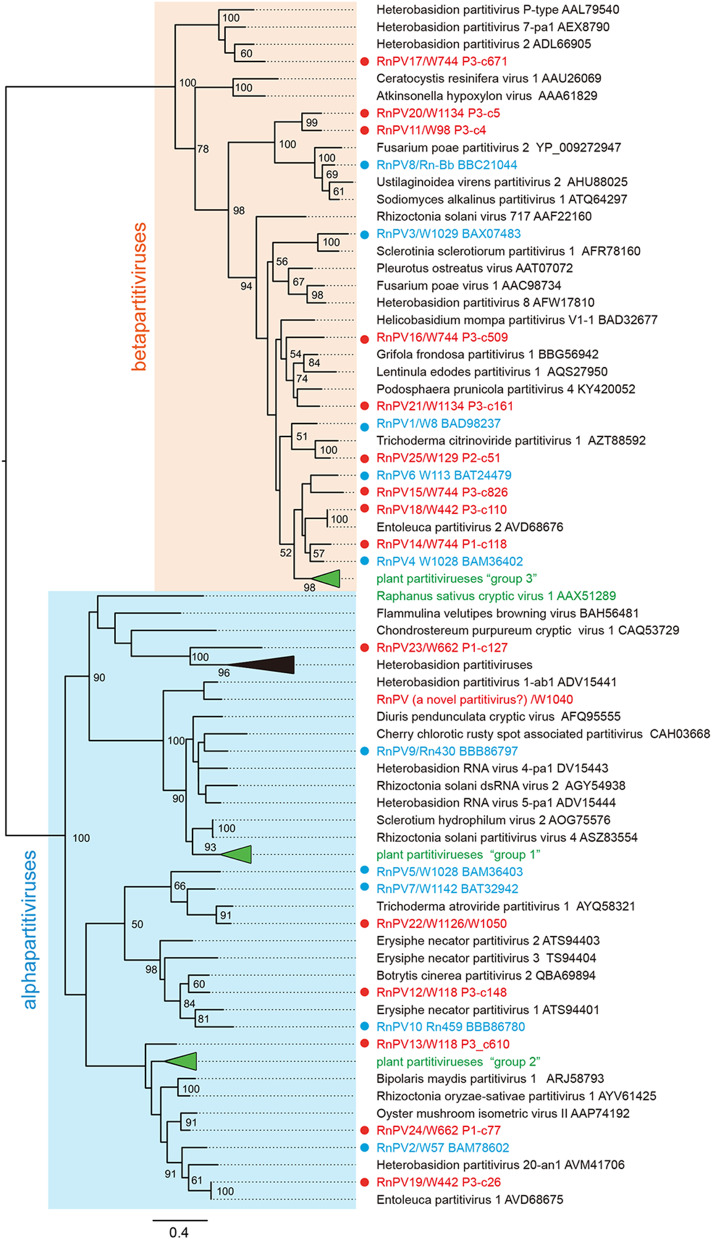
Phylogenetic analysis of novel partitiviruses from field-collected isolates of *R. necatrix*. Maximum likelihood (ML) phylogenetic trees based on amino acid alignments of RdRPs were constructed using PhyML 3.0 with a best fit model RtREV with +G +I +F. Sequences of members of *Alphapartitivirus* and *Betapartitivirus* genera were analyzed together with novel *R. necatrix* partitiviruses. Virus names are followed by GenBank accession numbers. Red and blue circles indicate novel or reported *R. necatrix* partitiviruses, respectively. The numbers at the nodes indicate bootstrap values. The virus names and accession numbers in the collapsed triangles are described in Supplementary Table S4.

Members of the *Alphapartitivirus* and *Betapartitivirus* genera have been characterized from 14 to 17 species of fungi (ascomycetous and basidiomycetous) and plants (largely dicot plants), respectively (Vainio et al., 2018a). Phylogenetic analysis based on RdRP, encoded by dsRNA1, of the novel 15 *R. necatrix* partitiviruses using selected alpha- and betapartitiviruses that included approved members of both genera revealed that six novel *R. necatrix* partitiviruses clustered with alphapartitiviruses with a bootstrap value of 100 (Figure 3). These included RnPV12, RnPV13, RnPV19, RnPV22, RnPV23, and RnPV24. The remaining nine novel *R. necatrix* partitiviruses (RnPV11, RnPV14, RnPV15, RnPV16, RnPV17, RnPV18, RnPV20, RnPV21, and RnPV25) were clustered with betapartitiviruses with bootstrap values of 100. Note that some inter-subgroup relationships within the alphapartitivirus branch were not well supported with high bootstrap values. Notably, most *R. necatrix* partitiviruses were discretely placed within the tree, while some viruses (RnPV4, RnPV6, RnPV14, RnPV15, and RnPV18) were clustered together and nested with a clade of plant betapartitiviruses (namely group 3) (Figure 3 and Supplementary Table S4).

### Phenotypic Effects of Novel Partitiviruses on Both Original and Experimental Hosts

The investigation of the biological properties of any virus requires comparing sets of isogenic virus-free and -infected strains. However, when assessing *R. necatrix*-partitivirus interactions, this was difficult because mixed infections occurred frequently and virus curing via hyphal tipping presented difficulties. To this end, we used a virion transfection approach now available for various virus/host combinations.

Of the 15 sequenced, novel partitiviruses of *R. necatrix* identified in this study, 13 (in 5 fungal strains) were tested for their effects on three virus-free strains of two fungal species: the standard strain, W97, of the original host species, *R. necatrix*, and two strains (EP155 wild-type and its Δ*dcl2* KO mutant, an antiviral RNA silencing deficient strain) of the experimental host, *C. parasitica*. Note that many of the fungal strains tested were co-infected by multiple partitiviruses (see Table 1). Obtained transfectants are summarized in Supplementary Table S3. RnPV11 was singly transfected in W97 (Table 2). The five, three and two viruses coinfecting W744, W118, and W1134, respectively, could not be separated via transfection of W97. No significant phenotypic change was observed in W97 transfectants, except W97 co-infected with five viruses (RnPV1 and RnPV14 to RnPV17) originally harbored in W744. Initially, the W97 transfectant infected by the five viruses displayed considerably reduced growth, its mycelia appeared to be deep white in color and were fluffy (Supplementary Figure S4A). Subsequent hyphal fusion with virus free W97 led to the presentation of a milder growth defect (Supplementary Figure S4C).

**Figure 4:**
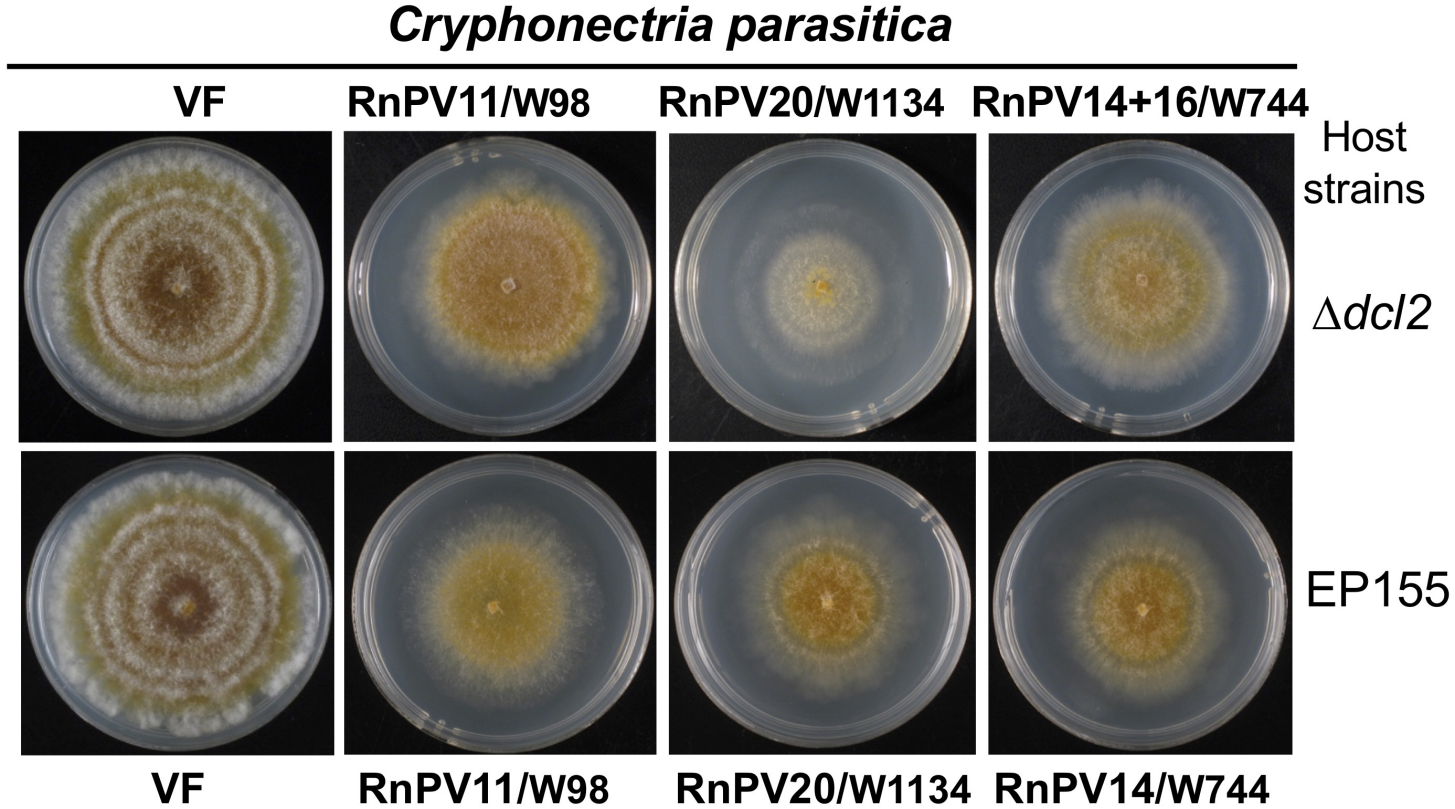
The morphology of *C. parasitica* colonies infected with betapartitiviruses from different *R. necatrix* fungal strains. *C. parasitica* Δ*dcl2* singly infected by RnPV11/W98 or RnPV20/W1134, and doubly infected by RnPV14, and RnPV16 from the W744 strain were grown in PDA for 1 week on a benchtop and photographed. *C. parasitica* EP155 colonies singly infected with the viruses are shown. VF refers to virus free strains.

Multiple partitiviruses, RnPV18 and RnPV19, coinfecting a single strain W442, segregated and their single infectants could be obtained in *C. parasitica* Δ*dcl2* (Supplementary Figure S4B). These Δ*dcl2* transfectants as well as other partitivirus transfectants (RnPV11, RnPV20 and RnPV14–16) (Supplementary Table S3) were then anastomosed with RNA silencing-proficient *C. parasitica* EP155 to move the viruses. Even after repeated coculturing, EP155 stably maintained partitiviruses (RnPV11, RnPV20, or RnPV14–16) and their infectants showed reduction in growth rates, pigmentation, and growth of aerial hyphae (Figure 4). On the other hand, EP155 strains singly infected by RnPV18 and RnPV19 were either infected asymptomatically or displayed only slightly reduced growth rates in EP155 (Supplementary Figure S4B). In Δ*dcl2*, most partitiviruses (RnPV11, RnPV20, RnPV14–16, and probably RnPV19) produced symptomatic infections and induced reduced growth rates and irregular margins (Figure 4 and Supplementary Figure S4B). Similar symptoms have previously been attributed to other partitiviruses from *R. necatrix*, i.e., RnPV2 and RnPV6 (Chiba et al., 2013a, 2016) (Table 2).

### Different Virus Accumulation Levels in Between Virus Strains and in Between Different Fungal Host Strains

**Figure 5:**
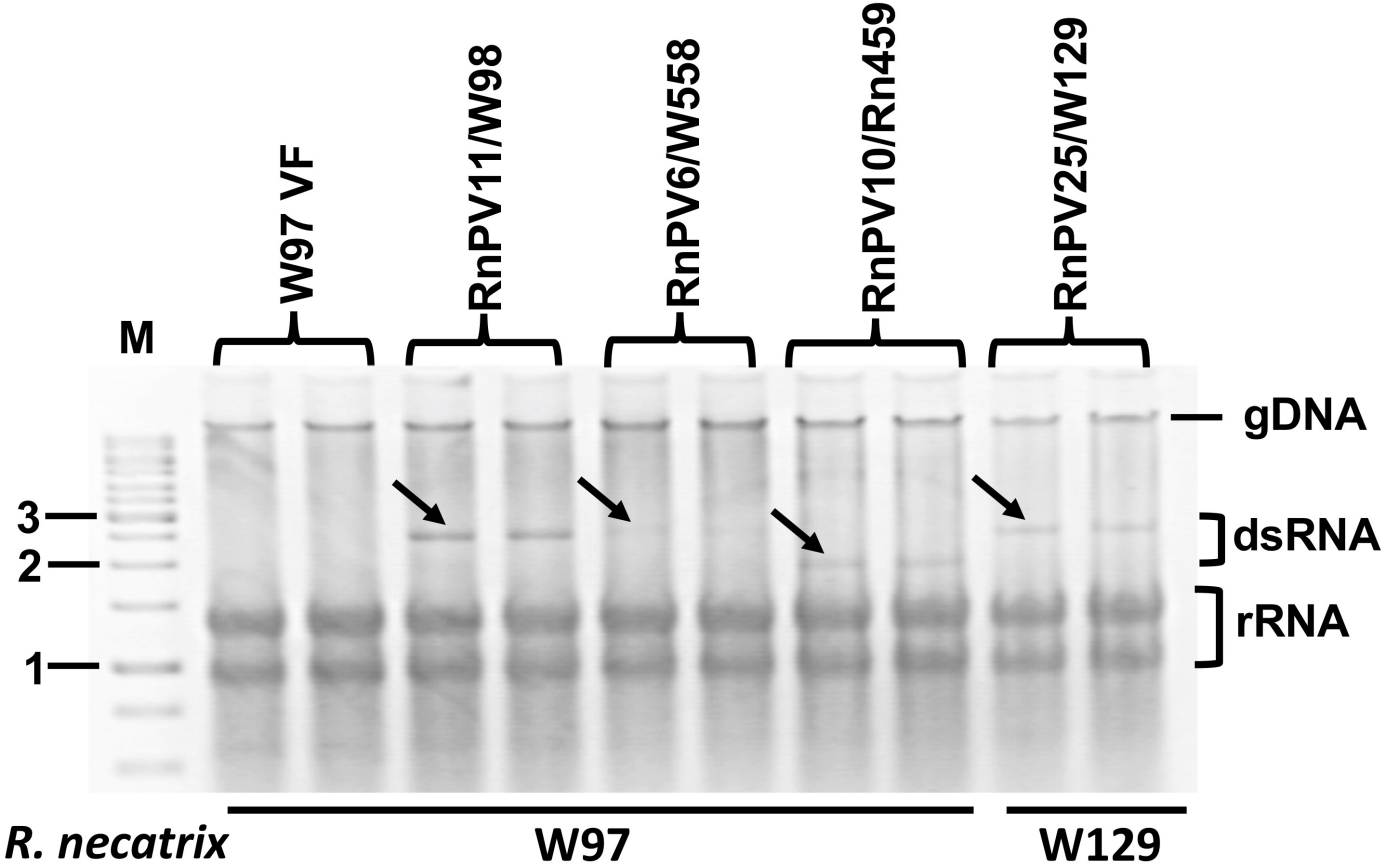
Virus accumulation of four partitivirus strains in *R. necatrix* host strains. One alphapartitivirus (RnPV10) and three betapartitiviruses (RnPV6, RnPV11, and RnPV25) were used for this analysis. Total nucleic acid fractions were isolated from W97 mycelia singly infected by RnPV11/W98, RnPV6/W558, or RnPV10/Rn549, and analyzed via 1.2% agarose gel electrophoresis. These W97 single infectants were obtained by transfection of protoplasts with respective partitivirus virions. For RnPV25/W129, the original host strain W129 was used. Black arrows indicate the migration positions of dsRNA genomic segments of the respective partitiviruses. M refers to the 1-kb DNA ladder. rRNA was used as a loading control. gDNA indicates the migration position of the host genomic DNA.

We attempted to compare genomic dsRNA accumulation in the standard *R. necatrix* strain W97. To this end, RnPV6/W558, RnPV10/Rn459, and RnPV11/W98 were selected, because their W97 single transfectants could readily be obtained. For RnPV25/W129, the original filed-collected strain W129 was used. Total RNA fractions were isolated from two biological replicates for each strain, and levels of viral dsRNA accumulation were compared by normalizing to host ribosomal RNAs (Figure 5). RnPV11/W98 dsRNA1 and dsRNA2 of 2.4 and 2.3 kbp accumulated in W97 at a level slightly greater than or comparable to, RnPV10/Rn459 (1.9 kbp + 1.9 kbp), or RnPV25/W129 (2.4 kbp + <2.0 kbp). The band intensity of RnPV6/W558 dsRNA (2.5 kbp + 2.5 kbp) was much fainter than those of dsRNA from the other three partitiviruses. Note that dsRNA1 and dsRNA2 of the four tested partitiviruses, except for RnPV25/W129, were completely sequenced and co-migrated in the agarose gel. Considering a second dsRNA band for RnPV25/W129, the size of partially sequenced dsRNA2 appears to be similar to dsRNA1 and have comigrated with it (Figure 5). These results suggest variability in partitiviral dsRNA accumulation within the same *R. necatrix* host strain.

**Figure 6:**
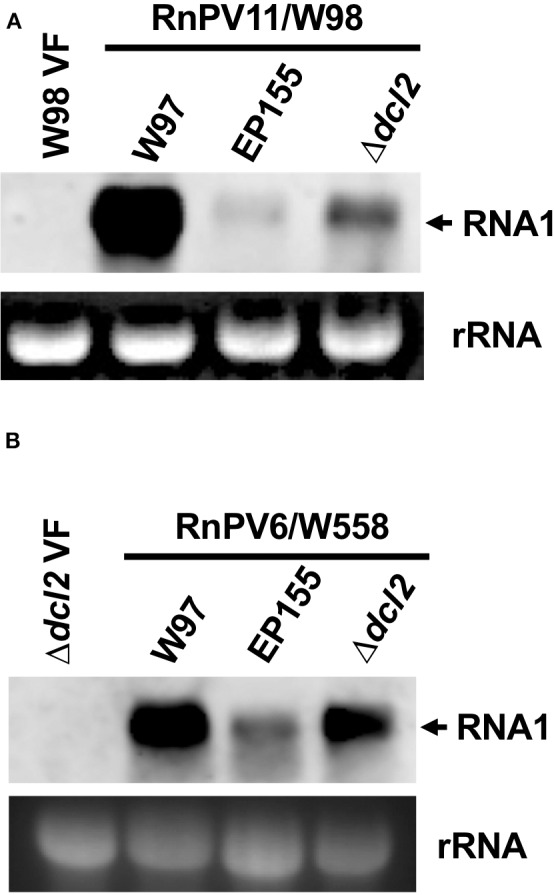
Comparison of virus accumulation in three different fungal strains *R. necatrix* W97 and *C. parasitica* wild-type EP155 and its mutant Δ*dcl2*. A. Equal amounts of total RNA was obtained from three fungal strains each singly infected by RnPV11/W98 **(A)** or RnPV6/W558 **(B)**, and subjected to Northern blotting. The probes used are DIG-labeled PCR fragments specific to RnPV11 dsRNA1 **(A)** and RnPV6 dsRNA1 **(B)**, respectively, that were prepared using specific primer sets, W98-RdRp-F and W98-RdRp-R, and W558-RdRp-F and W558-RdRp-R. See Supplementary Table S1 for the sequences of the primers used in probe preparation.

Generally, partitiviruses from *R. necatrix* accumulate to the greater levels in the original host, rather than the experimental host strain, *C. parasitica* (Chiba et al., 2013a, 2016). Next, we compared the accumulation of a single partitivirus (RnPV11 or RnPV6) in the three host strains, which included the standard *R. necatrix* strain W97, *C. parasitica* EP155 and Δ*dcl2*. Inspection of Figure 6 clearly shows that RnPV11/W98 and RnPV6/558 accumulated more in the RNA silencing-deficient Δ*dcl2* than in RNA silencing-competent EP155. However, both viruses accumulated more highly in W97 than in wild-type EP155 and Δ*dcl2*. We obtained a similar accumulation profile using three biological replicates (data not shown).

## Discussion

In this study, a total of 20 partitiviruses were isolated from 16 field isolates of the white root rot fungus, *R. necatrix*. The isolates were characterized molecularly and a subset was characterized biologically. Interestingly, 15 (RnPV11 to RnPV25) may belong to new species, Rosellinia necatrix partitivirus 11 to Rosellinia necatrix partitivirus 25, and six other partitiviruses (RnPV1, RnPV3–RnPV6) belong to known or previously proposed partitiviral species. This study has shown that all of the characterized partitiviruses should be placed within the two genera of *Alphapartitivirus* and *Betapartitivirus*, which are members of the family *Partitiviridae*. However, they show great variability with respect to their biological and virological properties. This study also provided a platform for the further exploration of molecular mechanisms that underly differences in viral symptoms and replication patterns.

The 15, completely sequenced, novel *R. necatrix* partitiviruses (6 alphapartitiviruses and 9 betapartitiviruses) provide interesting insights into partitivirus biology. Partitiviruses that infect fungi are currently classified into three genera: *Alphapartitivirus, Betapartitivirus*, and *Gammapartitivirus* (Nibert et al., 2014; Vainio et al., 2018a). This and previous studies have revealed the prevalence of alpha- and betapartitiviruses, but not gammapartitiviruses, in *R. necatrix* (Chiba et al., 2013a, 2016; Kondo et al., 2013b; Arjona-Lopez et al., 2018). Our failure to detect gammapartitiviruses in *R. necatrix* is surprising, because gammapartitiviruses have been reported only in ascomycetes thus far (Nibert et al., 2014; Vainio et al., 2018a). Among the other most extensively surveyed fungi for virus hunting are ascomycetous *C. parasitica* (class: Sordariomycetes), and *Sclerotinia sclerotiorum* (class: Leotiomycetes), and basidiomycetous *Heterobasidion* spp. The fact that no gammapartitiviruses have been reported from these fungi (Jiang et al., 2013; Vainio and Hantula, 2016) may suggest that gammapartitiviruses are restricted to specific ascomycetes and/or narrow host ranges, although a gammapartitivirus has been shown to replicate in plant protoplasts (Nerva et al., 2017b). *Alphapartitivirus* and *Betapartitivirus* each include both plant and fungal partitiviruses. It is presumed that those viruses, or their ancestors, may have been transferred between the two kingdoms, which eventually lead to their higher prevalence relative to gammapartitiviruses.

An interesting relation was found between the newly discovered betapartitivirus, RnPV21/W1134, and a previously characterized betapartitivirus, RnPV1/W8 (Sasaki et al., 2005), which was also discovered in the W744 strain in this study. The two segments of RnPV21 possessed the conserved terminal sequences, indicating that they represented the genome of the virus. However, the two viruses shared 56 and 94% amino acid sequence identities for RdRP and CP, respectively. While the highest degree of CP amino acid sequence identify was detected between RnPV1 and RnPV21, the highest degree of RdRP amino acid sequence identity (65%) was observed with an another betapartitivirus (Podosphaera prunicola partitivirus 4) from the sweet cherry powdery mildew fungus. Generally, partitivirus CP genes are less conserved than RdRP (Nibert et al., 2014). These facts strongly suggest that there might have been a reassortment event between two betapartitiviruses. Similar reassortment events have recently been proposed among isolates of different partitiviruses by Petrzik (2019).

Co-infection of the Japanese *R. necatrix* strain, W442, by two novel partitiviruses, RnPV18 (*Betapartitivirus*) and RnPV19 (*Alphapartitivirus*), was detected (Table 2). The Japanese fungal strain W442 was isolated in Hyogo Prefecture (Table 1). Interestingly, the same combination of partitiviruses (EnPV2 and EnPV1, closely related to RnPV18 and RnPV19, respectively) have been reported from Spanish isolates of *Entoleuca* sp. as well as *R. necatrix* that infested or colonized avocado soil of southern coastal area of Spain (Velasco et al., 2019, 2020). These observations suggest that interspecific horizontal transfer of the two partitiviruses may have occurred between *R. necatrix* and *Entoleuca* sp., as previously suggested or demonstrated for different viruses (Deng et al., 2003; Liu et al., 2003; Yaegashi et al., 2013; Vainio and Hantula, 2016; Khalifa and MacDiarmid, 2019). The coinfection of two fungal hosts by the same set of two taxonomically different partitiviruses (alpha- and betapartitiviruses) suggests the possibility of mutualistic or commensal interactions between the coinfecting partitiviruses. At present, we cannot rule out the possibility that the two viruses accidentally co-transferred from an originally co-infected fungal strain. Further, these partitiviruses were isolated from geographically distinct locations, Japan and Spain. The fact that RnPV18 and RnPV19, and EnPV2 and EnPV1 were isolated from two different sympatric fungal species suggests that contamination in the laboratory was highly unlikely. It is difficult to determine the origin of the fungus carrying the viruses, which may have been brought together via crop plants and/or host fungi as a result of human activities such as tourism and trade (see below).

Horizontal transfer of fungal viruses between two strains of single fungal species, and also between different fungal species, have been suggested by several research groups (Yaegashi et al., 2013). The same subset of partitiviruses, megabirnaviruses (dsRNA viruses), hypoviruses, and fusagraviruses (ssRNA viruses) were detectable within two different fungal species, *R. necatrix* and *Entoleuca* sp., collected from avocado fields in Andalusia, Spain (Velasco et al., 2018, 2019). Previous viral identification and characterization studies compared genomic sequences of viruses that belonged to the same species, but were isolated from different continents. For example, 95 and 94% amino acid sequence identities were detected between the isolates of positive-sense (a mitovirus, family *Narnaviridae*) and negative-sense (a mymonovirus, family *Mymonavirudae*) ssRNA viruses from *Sclerotinia sclerotiorum* collected from the USA and Australia (Mu et al., 2017). The sequence identities determined in this study were greater than those previously reported. This may suggest that the virus-harboring fungi were imported relatively recently with crop seedlings. In Spain, *R. necatrix* is an endemic soil-borne inhabitant in soils in which previous rainfed susceptible crops such as olive, almond, and grapes were cultivated. Later (1970's) these crops were replaced by high-value avocado irrigated crop (Lopez-Herrera and Zea-Bonilla, 2007), in which soils are highly infested by this pathogen with some colonized by the antagonistic fungi *Entoleuca* sp. (Arjona-Girona and Lopez-Herrera, 2018). EnPV1 and EnPV2 and these have been also detected in a *Fusarium* isolate collected from the same avocado orchards in addition to *Entoleuca* sp. and *R. necatrix* (Velasco et al., 2019, 2020). Presumably, these fungal viruses have been present in these soils for many years and their horizontal transmission between different fungal species inhabiting the same soils is likely to happen. However, it remains unknown which of them has been the initial source of transmission of these fungal viruses to the rest of fungal species or how the viruses have been horizontally transferred.

Of the 16 fungal isolates tested, five produced mixed infections, as exemplified in W744 that was infected with five different betapartitiviruses. There have been reported cases of virus/virus interactions in mixed infections (Hillman et al., 2018). Thus, there is a need to obtain single infectants when assigning two genomic segments to single viruses and characterizing each partitivirus. To this end, we used the transfection approach previously developed for assessing various virus-host combinations (Sasaki et al., 2006; Kanematsu et al., 2010; Chiba et al., 2013a, 2016; Salaipeth et al., 2014). We obtained transfectants either in the *R. necatrix* W97, *C. parasitica* EP155 genetic background, or both W97 and EP155, which were singly infected partitiviruses. For these viruses, we confirmed that the two segments matched that of the genome of specified partitiviruses. For other coinfecting partitiviruses, our conclusions were based on the highly conserved terminal sequences found between segments (Supplementary Table S2). Furthermore, this facilitated the comparison of virus titers in EP155 and its Δ*dcl2* disruptant.

Some of the characterized partitiviruses differed from one another with respect to their accumulation levels and degree of symptom induction (Figures 4–6 and Table 2). These characteristics are related to antiviral/counter-defense responses incited by hosts and viruses. Previous studies have revealed that many homologous and heterologous viruses are targeted by antiviral RNA silencing (Segers et al., 2007; Sun et al., 2009; Chiba et al., 2013a,b, 2016; Salaipeth et al., 2014; Chiba and Suzuki, 2015). Many of these viruses induce antiviral RNA silencing via transcriptional up-regulation of the genes including a dicer (*dcl2*) and an argonaute (*agl2*) (Sun et al., 2009; Chiba and Suzuki, 2015). Previous reports showed that a victorivirus (dsRNA virus, family *Totiviridae*), RnVV1, was sensitive to, and targeted by, RNA silencing, but did not induce antiviral RNA silencing (Chiba and Suzuki, 2015). CHV1 is tolerant to RNA silencing and does not induce *dcl2* transcription, which was largely attributed to the activity of the RNA silencing suppressor, p29 (Segers et al., 2007; Chiba and Suzuki, 2015). Andika et al. (2017) developed an antiviral RNA silencing monitoring system through the *dcl2* transcription using a GFP reporter construct driven by the *dcl2* promoter with *C. parasitica* genetic background. Interestingly, partitiviruses isolated from *R. necatrix* showed great variability in induction levels of *dcl2* (Aulia et al., unpublished data). It is known that some partitiviruses tolerate RNA silencing while others are sensitive to silencing (Chiba et al., 2016) (Chiba and Suzuki, unpublished data). This study provided researchers with the materials necessary to obtain insights into mechanisms controlling diversity of host-partitivirus interactions.

## Data Availability Statement

The datasets presented in this study can be found in online repositories. The names of the repository/repositories and accession number(s) can be found here: https://www.ddbj.nig.ac.jp/, LC517370-LC517399, LC521312-LC521313 (32 entries).

## Author Contributions

NS designed the experiments and wrote the manuscript. PT, SH, CM, KH, and JA-L performed the experimental work. SK collected samples. PT, SK, CL-H, HK, and NS analyzed the data. PT and HK were involved in discussion and manuscript revision. All authors have given approval to the final version of the manuscript.

## Conflict of Interest

The authors declare that the research was conducted in the absence of any commercial or financial relationships that could be construed as a potential conflict of interest. The reviewer EV declared a past co-authorship with one of the authors NS.

## Footnotes

^1^https://nucleobytes.com/enzymex/index.html

^2^http://tree.bio.ed.ac.uk/software/
