# Supplementary material for: Diverse Partitiviruses From the Phytopathogenic Fungus, Rosellinia necatrix
Source: Front Microbiol. 2020 Jun 26;11:1064. doi: 10.3389/fmicb.2020.01064 (PMC7332551; doi:10.3389/fmicb.2020.01064)
Supplement: Supplementary file 1 [file Data_Sheet_1.pdf]

Supplementary information for

Diverse partitiviruses from the phytopathogenic fungus, *Rosellinia necatrix*

Paul Telengech[1], Sakae Hisano[1], Cyrus Mugambi[1], Kiwamu Hyodo[1], Juan Manuel Arjona-López[1,2], Carlos José López-Herrera[2,] Satoko Kanematsu[3,4], Hideki Kondo[1], and Nobuhiro Suzuki[1+]

[1] Institute of Plant Science and Resources, Okayama University, Chuo 2-20-1, Kurashiki, 710-0046, Japan

[2] Institute for Sustainable Agriculture, Spanish Research Council. Alameda del Obispo s/n 14004. Córdoba, Spain

[3] Institute of Fruit Tree Science, National Agriculture and Food Research Organization (NARO), Morioka, Iwate 020-0123, Japan Institute of Fruit Tree Science (NIFTS), National Agriculture and Food Research Organization (NARO), Morioka, Iwate 020-0123, Japan

[4] Present address: NIFTS, NARO, Tsukuba, Ibaraki, 305-8605, Japan.

Running title: Diverse partitiviruses of *Rosellinia necatrix*

+Correspondence may be sent to N. Suzuki
Agrivirology Laboratory, Institute of Plant Science and Resources, Okayama University
Kurashiki, Okayama 710-0046, JAPAN
Telephone: 81-86-434-1230
FAX: 81-86-434-1232
E-mail: nsuzuki@okayama-u.ac.jp

Supplementary FIGURE S1. The presence of a potential translational enhancer (CAA repeats) at the 5′ UTR of three *R. necatrix* partitiviruses, RnPV19, RnPV22 (alphapartitiviruses) and RnPV20 (a betapartitivirus).

Supplementary FIGURE S2. Conserved sequences at the 5′ plus strand termini of novel *R. necatrix* alpha- and betapartitiviruses. Conserved residues are highlighted in grey and the consensus is shown (M: A or C; N: any nt; S: C or G; W: A or U; Y: C or U).

Supplementary FIGURE S3. Phylogenetic ML trees and a pairwise comparison of RdRPs (dsRNA1) and CPs (dsRNA2) encoded by *R. necatrix* alphapartitiviruses (A) and betapartitiviruses (B). Names of novel partitiviruses (RnPV11–25) are indicated using red text. Each number (selected) and/or colored box within the matrix represents pairwise amino acid identities (%) between partitiviruses that were calculated using SDT version 1.2.

Supplementary FIGURE S4. Colony morphology of *R. necatrix* W97 and *C. parasitica* wild-type EP155 and its mutant Δ*dcl2* infected with partitiviruses. (A) Phenotypes of *R. necatrix* W97 uninfected (VF) and quintuply infected with RnPV1 + RnPV14 to RnPV17 (originally harbored in W744). (B) Phenotype of *C. parasitica* strains Δ*dcl2* and EP155 infected with RnPV18/W442, RnPV19/W442 and RnPV3/W118. (C) Colony morphology of *R. necatrix* W97 infected with the different partitiviruses. Purified virion fractions from W98, W118, W442, W1134, and W774 were transfected into W97, mostly resulting in mixed infection by more than two partitiviruses. The virus-infected W97 colonies, in parallel with virus free W97 strain, were grown in PDA for one week on a benchtop and photographed. W97 infected by RnPV1 + RnPV14 to RnPV17 was a subculture from the original transfectant shown in (A).

Supplementary Figure S1

Virus		CAA repeats
RnPV19	dsRNA1	GAAGACUCCGACAGCUCU**CAA**CCGCUCUC-----------GAUUCC**CAA**CUUAAUUGUCU
	dsRNA2	AGAA-----GACUCCUUCUACCUGCUUACAC**CAA**GUAAAAGAUUCC**CAA**CC**CAA**UUGUCU
		..*. ** * **...* *.***. * **********..********
	dsRNA1	GCUCUCCGUCUUAAGAACUUCCCUACGU**CAA**UUUUCUCUCGUA**CAA**GCUACUCUUAUAAA
	dsRNA2	GCUCUCCGU**CAA**AAGAACUUCCCUAUU**CAA**ACUUUCUG**CAA**UA**CAA**GCUACUCUUUUAA-
		********** *************. . **.***** . .************** ***
RnPV20	dsRNA1	GAATACTGACTG**CAA**GCACCTCGCT---CG**CAA**ACT**CAA**TTCTGATCA**CAA**TGATTT**CAA**
	dsRNA2	GAATACTGCCTG**CAA**GCACCCCCCTCTG**CAA**AATCG**CAA**TTCTGATCA**CAA**ACTCCA**CAA**
		******** ***********.* ** *. ** * ************** .. ***
	dsRNA1	TTTTCG**CAA**TTGTTCTCATGCACTT**CAA**GCT**CAA**ACTCTGGGCTAACTCTCTGCGCCACG---
	dsRNA2	TTTTCTC-----------------T**CAA**---CGAAATCTAAC**CAA**TTTCCTTGCGT**CA**--**A**AC
		***** * **** *.** ***.. * * .**..****.*
RnPV22	dsRNA1	AGAACCTTAACGGACGAATTGCACGAGAGCTTA-CACT**CAA**CCGTT**CAACAA**CTA**CAA**TT
	dsRNA2	AGAACCTTAACGGACGAATTGCACGAGAGC**CAA**CCACT**CAA**CCGTT**CAACAA**CTA**CAA**TT
		*****************************. * *************************
	dsRNA1	CGACACACTAGGACCTAAAGCCA**CAA**GATGACAGAGAATCTAACC----TACGT**CAA**ATA
	dsRNA2	CGACACACTAGGACCTAAAGCGA**CAA**GACT**CAACAACAA**TCAGACATGTTTTCTCGATCG
		******************* ******. * *. * ..*. * * . **.* ..

Supplementary Figure S2

Alphapartitiviruses

```
RnPV12-dsRNA1  CGAAAACUUAACCAUAGCUUU
RnPV12-dsRNA2  CGAAAACUUAACCAUAGUCUU
RnPV19-dsRNA1  -GAAGACUCCGACAGCUCUCA
RnPV19-dsRNA2  AGAAGACUCCUUCUACCUGCU
RnPV22-dsRNA1  AGAACCUUAACGGACGAAUUG
RnPV22-dsRNA2  AGAACCUUAACGGACGAAUUG
   CONSENSUS    GAANMYUNMNNSWNNNNNYN
```

Betapartitiviruses

```
RnPV11-dsRNA1   GAAUCCUCGAUAUAGCGACU
RnPV11-dsRNA2   GAAUCCUCGAUAUACCGACU
RnPV14-dsRNA1   GGUUUUCUCUAGGAAAGUCA
RnPV14-dsRNA2   GGUUUUCUCUAGGAAAGUCA
RnPV15-dsRNA1  AGUAUUUCUCUGCUCCGCCCC
RnPV15-dsRNA2  AGUAUUUCUCUGCUCCGCCCC
RnPV16-dsRNA1  UGAUUUUCUUGCAGGCCCCCC
RnPV16-dsRNA2  UGAUUUUCUUGCAGGCCCCCC
RnPV17-dsRNA1   GAAUAAGGUUCUAUACGCCC
RnPV17-dsRNA2   GAAUAAGGUUCUAUACGCCC
RnPV18-dsRNA1   GGUUUUCUCUAGCUCGCGCC
RnPV18-dsRNA2   GGUUUUCUCUAGCUCGCGCC
RnPV20-dsRNA1   GAAUACUGACUGCAAGCAAA
RnPV20-dsRNA2   GAAUACUGCCUGCAAGCAAA
RnPV21-dsRNA1  -GACUUUUCGUAUAGCCCGCC
RnPV21-dsRNA2  GGACUUUUCGUAUAGCCCGCC
  CONSENSUS     GNNUNNNNNNNNNNNNSNMN
```

Supplementary Figure S3

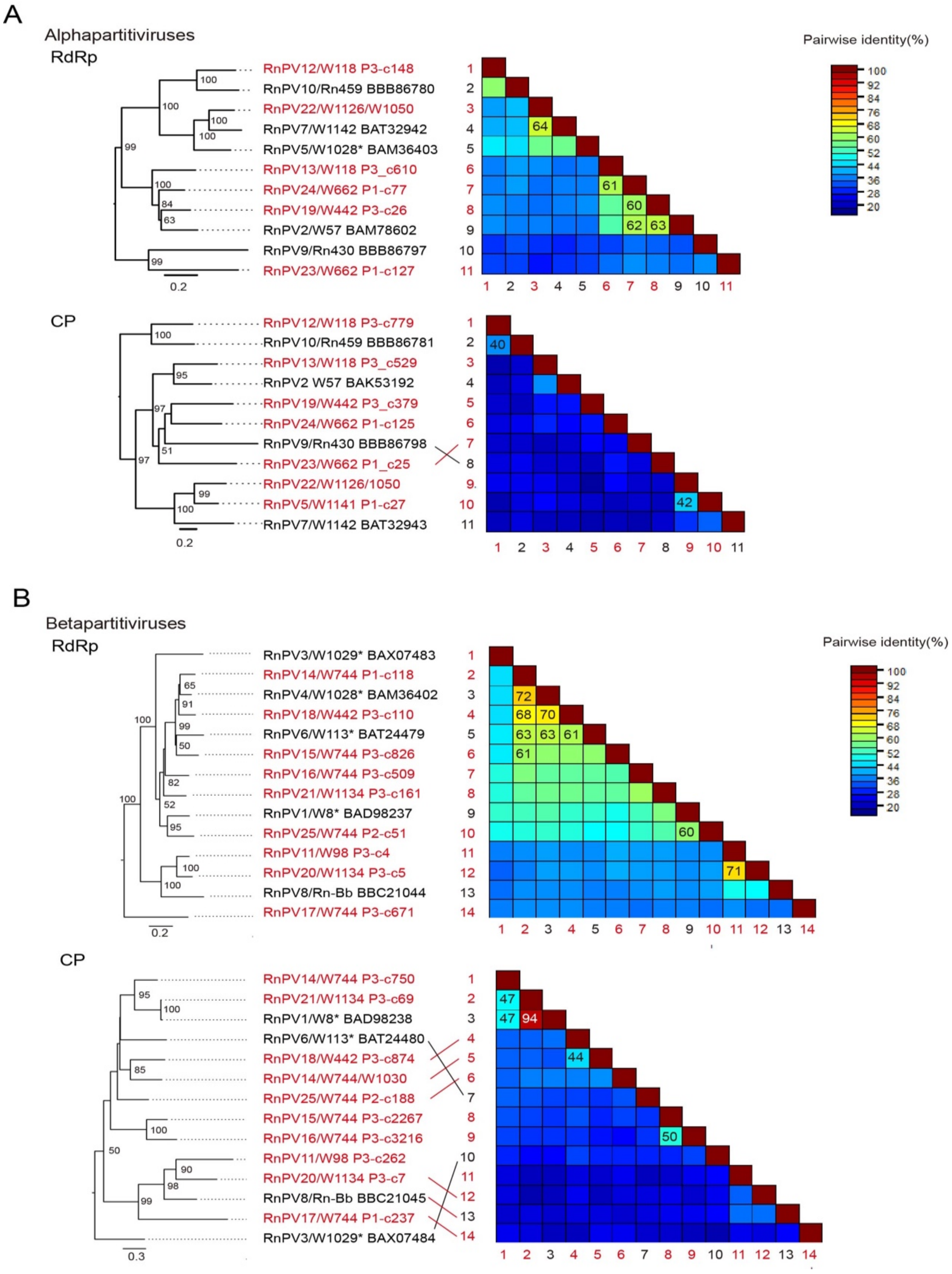

Supplementary Figure S4

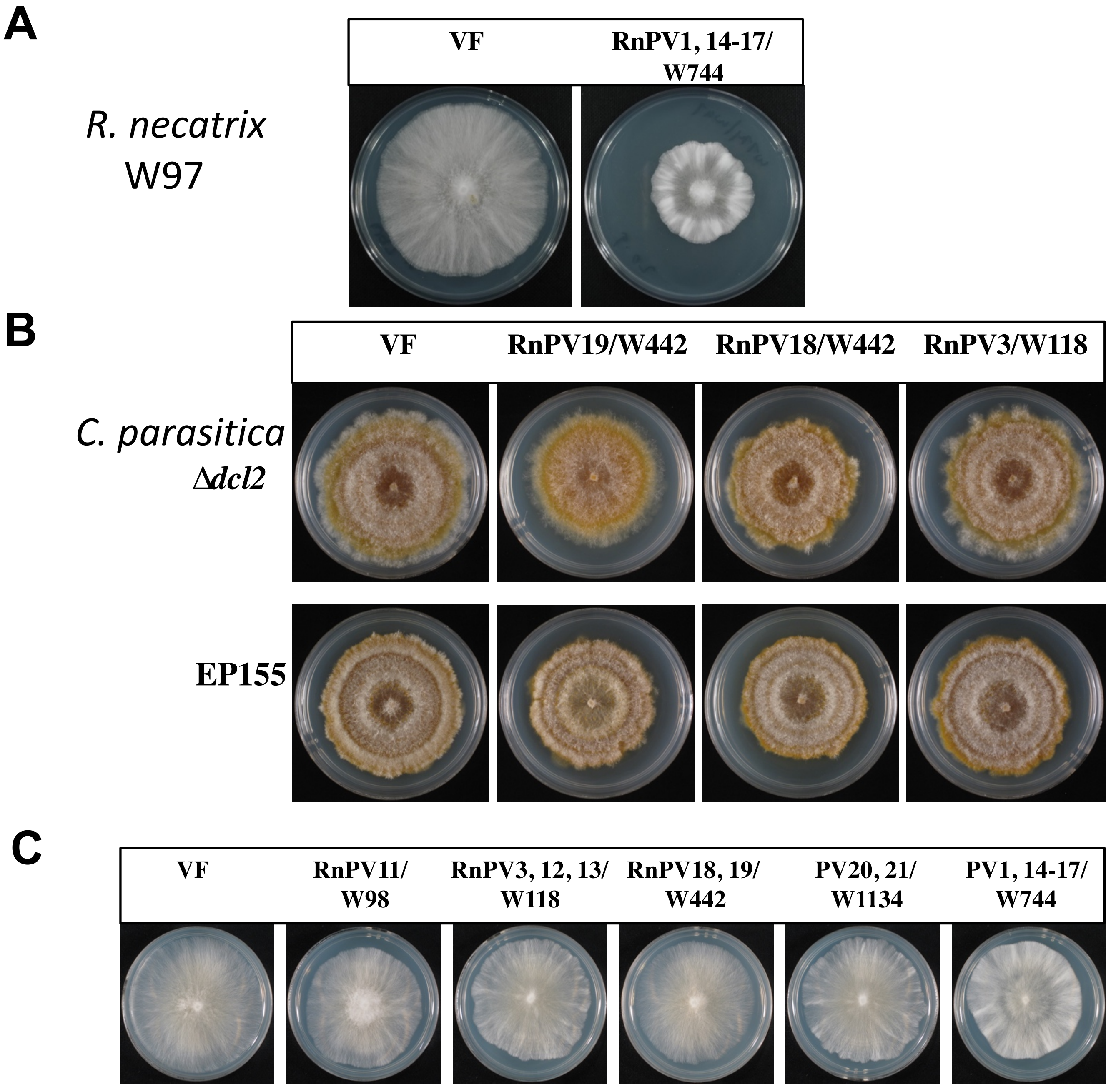

Supplementary Table S1. List of primers

No	PRIMER NAME	PRIMER SEQUENCE	PURPOSE	No	PRIMER NAME	PRIMER SEQUENCE	PURPOSE
1	ngs 262R 98 cp	ACGGAGATAGGCGTCAC AGT	RT-PCR	23	ngs 509R rd w744	AATGTAGCATCGGTTGTTCC G	RT-PCR
2	ngs 262F 98 cp	ATGGACACTTCCGCATC TGG	RT-PCR	24	ngs 509F rd w744	CTGCTGGTTCTGACCGAAC	RT-PCR
3	ngs 779R cp w118	TGCAGGACGGGATGTAG TTC	RT-PCR	25	ngs 2267R cp w744	GAATGAAGGTTACGTCGTT GTTG	RT-PCR
4	ngs 779F cp w118	CGTGTCTATGACGATAC AGAG	RT-PCR	26	ngs 2267F cp w744	TAATTGGAACAGAGCGTCC GTA	RT-PCR
5	ngs 148R Rd w118	TATCGCCTTCTGAGCGT AAGT	RT-PCR	27	ngs 118R Rd w744	ATATCACCTGGGATATAGG ATTCT	RT-PCR
6	ngs 148F Rd w118	TATACGATACTTGCGTC GAGC	RT-PCR	28	ngs 118F Rd w744	CCCGAACACGGTATCAACT ATAA	RT-PCR
7	ngs 610R Rd w118	GTAGCGTAGGGAGCACC AAT	RT-PCR	29	ngs 750R cp w744	AGACGACGTTTGACATAAG CG	RT-PCR
8	ngs 610F Rd w118	GTCCTGCTTGCTCAACT ACG	RT-PCR	30	ngs 750F cp w744	TCGGAAGCGCATTGATGTC AA	RT-PCR
9	ngs 529R cp w118	GGTAGTCAGGGCAGTTT CTC	RT-PCR	31	ngs 826R Rd w744	GGTCGACAGTATTTGTCTA GGA	RT-PCR
10	ngs 529F cp w118	CGATTGCTCTCCCTCTGG AA	RT-PCR	32	ngs 826R Rd w744	AAGTACCGCACAATGTCCG C	RT-PCR
11	ngs 379R cp w442	GAGTTGGCGTCGTTCTC ATC	RT-PCR	33	ngs 127 rd F	AGATTATCGCTGACGCTCG C	RT-PCR
12	ngs 379F cp w442	ACTGCTCAATCGATGGT CCTT	RT-PCR	34	ngs 127 rd R	CAGACCAGATAAGATGCCG C	RT-PCR
13	ngs 110R Rd w442	GGATGTAGGATTCAGCA GCAT	RT-PCR	35	ngs 77 rd F	TGTTGGCGCTCCTTACGCTA	RT-PCR
14	ngs 110F Rd w442	ACTCACTCTCGACATGA AACG	RT-PCR	36	ngs 77 rd R	TGTGTGTCTAGCGCGTCGAT	RT-PCR
15	ngs 5R Rd w1134	GTTCTACAAAGGCAACG TGTG	RT-PCR	37	ngs 51 rd F	CTGGTCACTCTATGCATCCA ATT	RT-PCR
16	ngs 5F Rd w1134	CCTCAGCTGACGTACAC GTT	RT-PCR	38	ngs 51 rd R	TCAACAGCATATACAGGGC GTA	RT-PCR
17	ngs 7R cp w1134	AGAAGGACGAGAAGAC GTTGT	RT-PCR	39	ngs 43 rd F	TCGTAATCACATCTCAGATC GAG	RT-PCR
18	ngs 7F cp w1134	ACTCGTTGGCACAAACT ACGT	RT-PCR	40	ngs 43 rd R	GAGTTGTCGTCTCCCATTAT GAA	RT-PCR
19	ngs 161R rd w1134	TGTTGAGTCGTTTGTGG ACGT	RT-PCR	41	ngs 497 cp F	ACACCGTTCTTCCACCGATC	RT-PCR
20	ngs 161F rd w1134	TATGCTATCCCGAACGT GGC	RT-PCR	42	ngs 497 cp R	TGATGCGGAGGTGTTGGAA G	RT-PCR
21	ngs 3216R cp w744	ACGATGGGAGTAGCCAG CA	RT-PCR	43	w118-5177 Rd F	CACCTCTGACGAGTATGAC G	RT-PCR
22	ngs 3216F cp w744	CTCTTCGCTTCTATGGTT CTG	RT-PCR	44	w118-5177 Rd R	GTCGGATTTGACGACAGCA G	RT-PCR

No	PRIMER NAME	PRIMER SEQUENCE	PURPOSE	No	PRIMER NAME	PRIMER SEQUENCE	PURPOSE
1	610F	GGTGACGTAGTTGAGCAAGC	RLM-RACE	24	148R	TTACCGTCGAAGCAAGACC	RLM-RACE
2	610R	CGATTAGTATTTGGCGCTCCTT	RLM-RACE	25	26F	ATGATCGAGAGTGGGAAGCAA	RLM-RACE
3	379F	GGAGCCGGAGCCAATCCAA	RLM-RACE	26	26R	TCCGACACTCGAATGACTAAG	RLM-RACE
4	379R	GCTCGATGAGAACGACGCC	RLM-RACE	27	874F	GTTAGAGGTCTTGGTCATACC	RLM-RACE
5	826F	GCAGAGAGTACCATATTCCAG	RLM-RACE	28	874R	GTTAACGCTGTCTACGCTACT	RLM-RACE
6	826R	GAAGCCGCCCGAATACTGAT	RLM-RACE	29	671F	TGTACTTCGCGAGAGTGACCA	RLM-RACE
7	2267F	GAGCAGTTGGGTAGTAGACGA	RLM-RACE	30	671R	GTGGTATGCAAGAACTCAATCGC	RLM-RACE
8	2267R	TCCGCTCTCGGATATTCCTTC	RLM-RACE	31	4F	TAGACCATGTAGCAGAGCTGA	RLM-RACE
9	509F	CAGACGCATAAGCGAGTCCAAT	RLM-RACE	32	4R	CAGTCTACGCTGTCGCTCT	RLM-RACE
10	509R	CTTCAACGAATATCCCACGCTC	RLM-RACE	33	237F	GTGTGAAGCATCGTAACGAGG	RLM-RACE
11	262F	TTGCGGATTGCGAACCACTGT	RLM-RACE	34	237R	TCTCGTCCAGAACTGGCTTTC	RLM-RACE
12	262R	TGCCCTCTACACCGACAACA	RLM-RACE	35	110F	GCGGCCAATATCATAGGGAC	RLM-RACE
13	779F	GATCCGAGGTTGCAGATTGGA	RLM-RACE	36	110R	ACACAGTATGCTAAGCTCCCG	RLM-RACE
14	779R	GTCGCTATCTCCTCAATTGTCC	RLM-RACE	37	7F	AGTTGAGAGCATGCGAGACGT	RLM-RACE
15	3216F	TCCGAGCTGTGGAACGATCAT	RLM-RACE	38	7R	CGTCACCAATGTCACCTTCAAC	RLM-RACE
16	3216R	ATGAAGCTCGCCTCCGCAAG	RLM-RACE	39	5F	GTGTACTTGATAACGCGAGTGA	RLM-RACE
17	347F	TGCGACGTTCGGCGAAGATT	RLM-RACE	40	5R	AATCCGACCCGTTTACGCC	RLM-RACE
18	347R	CCACTCCTATGCTCATCAACTCA	RLM-RACE	41	69R	CGAACGCGTTGATTGGGTAG	RLM-RACE
19	750F	GCGGGTCGGTGACATTATCTT	RLM-RACE	42	69F	TCTCTACCTCATTGACTTCGCT	RLM-RACE
20	750R	CTCAAGATCGTGCTCTCATCC	RLM-RACE	43	W558-RdRp-F	TACGCCCTGTTTACGCCGT	Probe preparation
21	161F	GGACCGGAGTACAGTAATGAC	RLM-RACE	44	W558-RdRp-R	ATGTGATAACGAGTGAGAGCGT	Probe preparation
22	161R	AGGAACTGGCTATCACAACCG	RLM-RACE	45	W98-RdRp-F	GCCCTCAACTTTCAATCCTTTG	Probe preparation
23	148F	AGCTGCTGATAATGAGGTGAG	RLM-RACE	46	W98-RdRp-R	TACGCTTCTACGCGCCATG	Probe preparation

Supplementary Table S2. Conserved 5′ and 3′ genome sequences of dsRNA1 and dsRNA2 of *R. necatrix* partitiviruses

VIRUS	5'-TERMINAL SEQUENCE	3'-TERMINAL SEQUENCE
RnPV10	dsRNA1 **AGATTTAAGT**CCCTCCGGAA---<br>dsRNA2 **AGATTTAAGT**CCCTCCGGAA---<br>********************	dsRNA1 ---TTTCCTAATGTTAAAGC<br>dsRNA2 ---TTTCCTTCATTTTTAAGC
RnPV11	dsRNA1 **GAAUCCUCGA**UAUAGCGACUCCCUCG---<br>dsRNA2 **GAAUCCUCGA**UAUACCGACUCCC-CG---<br>************** ******** **	Poly (A) with no additional nucleotides
RnPV12	dsRNA1 **CGAAAACUUAA**CCAUAGCUUUCGAUCUU---<br>dsRNA2 **CGAAAACUUAAC**CAUAGUCUUCGAUCUU---<br>*****************..*********	dsRNA1 ---CAAAAAAAAAAACC<br>dsRNA2 ---CAAAAAAAAAAAAACC
RnPV13	dsRNA1 – 5′ terminal incomplete	dsRNA1 ---AAAAAAAAAAAAUUU<br>dsRNA2 ---AAAAAAAAAAAAUUUU
RnPV14	dsRNA1 **GGUUUUCUCU**AGGAAAGUCACU---<br>dsRNA2 **GGUUUUCUCU**AGGAAAGUCACU---<br>**********************	Poly (A) with no additional nucleotides
RnPV15	dsRNA1 **AGUAUUUCUC**UGCUCCGCCCCUCGCGCCCA---<br>dsRNA2 **AGUAUUUCUC**UGCUCCGCCCCUCGCGCCCA---<br>******************************	Poly (A) with no additional nucleotides
RnPV16	dsRNA1 **UGAUUUUCUU**GCAGGCCCCCCCGCUCCCAA---<br>dsRNA2 **UGAUUUUCUU**GCAGGCCCCCCCGCUCCCAA---<br>******************************	dsRNA1 ---AAAAUAUCU<br>dsRNA2 ---AAAAUAUCUC
RnPV17	dsRNA1 **GAAUAAGGUU**CUAUACGCCCG---<br>dsRNA2 **GAAUAAGGUU**CUAUACGCCCG---<br>*********************	dsRNA1 ---AAAAAAAAAAAAUCC<br>dsRNA2 ---AAAAAAAAAAAAUC
RnPV18	dsRNA1 **GGUUUUCUCU**AGCUCGCGCCUCA---<br>dsRNA2 **GGUUUUCUCU**AGCUCGCGCCUCA---<br>***********************	dsRNA1 ---AAAAAAAAAUAUCC<br>dsRNA2 ---AAAAAAAAAUUUCCA
RnPV19	dsRNA1 –**GAAGACUCC**GACAGCUCUC---<br>dsRNA2 A**GAAGACUCC**UUCUACCUGC---<br>********* *. *.. *	dsRNA1 ---AAAAAAAAAAAAGU<br>dsRNA2 ---AAAAAAAAAAAAGU
RnPV20	dsRNA1 **GAAUACUG**ACUGCAAGCA---<br>dsRNA2 **GAAUACUG**CCUGCAAGCA---<br>******** *********	dsRNA1 ---CAAAACCCAAAAAAAAAACCC<br>dsRNA2 ---CCAAACCCAAAAAAAUACCCC
RnPV21	dsRNA1 –**GACUUUUCGU**AUAGCCCGCCC---<br>dsRNA2 G**GACUUUUCGU**AUAGCCCGCCC---<br>*********************	dsRNA1 ---AAAAAAAAAAGUC<br>dsRNA2 ---ATTACCAAAAGGUC
RnPV22	dsRNA1 **AGAACCUUAA**CGGACGAAUUGCACGAGAGC---<br>dsRNA2 **AGAACCUUAA**CGGACGAAUUGCACGAGAGC---<br>******************************	dsRNA1 ---AAAAAUAAA<br>dsRNA2 ---AAAAAUAAA

Supplementary Table S3. Partitiviruses introduced into *R. necatrix* W97, *C. parasitica* EP155, and *C. parasitica* Δ*dcl2*

VIRUS STRAIN (ABBREVIATION)	HOST STRAIN	VIRUS TRANSFECTED		
		W97	Δ *dcl2*	EP155
RnPV11	W98	✓	✓	✓
RnPV12	W118	✓	-	-
RnPV13		✓	-	-
RnPV3-W118		✓	✓	✓
RnPV18	W442	✓	✓	✓
RnPV19		✓	✓	✓
RnPV14	W744	✓	✓	✓
RnPV15		✓	-	-
RnPV16		✓	✓	-
RnPV17		✓	-	-
RnPV1-W744		✓	-	-
RnPV20	W1134	✓	✓	✓
RnPV21		✓	-	-
RnPV6-W558	W558	✓	✓	✓

✓ : transfectant obtained
-: experimental introduction not attempted

Supplementary Table S4. Plant fungal partitiviruses included in the phylogenetic tree

	Virus name	Accession No.
**Plant partitivirus group 1**		
	Beet cryptic virus 1	ACA81389
	White clover cryptic virus 1	AAU14888
	Carrot cryptic virus	ACL93278
	Vicia cryptic virus	AAX39023
	Dill cryptic virus 1	AGY36136
	Red clover cryptic virus 1	AGY36138
**Plant partitivirus group 2**		
	Arabidopsis halleri partitivirus 1	BAV56959
	Rose partitivirus	ANQ45203
	Pear alphapartitivirus	BBA66577
	Vicia faba partitivirus 1 ds3	ABJ99996
	Medicago sativa alphapartitivirus 1	AXY93817
	Medicago sativa alphapartitivirus 2	QBC36014
	Raphanus sativus partitivirus 1	ALT00589
**Plant partitivirus group 3**		
	Cannabis cryptic virus	AET80948
	Crimson clover cryptic virus 2	AGJ83769
	Dill cryptic virus 2	AGJ83771
	Hop trefoil cryptic virus 2	AGJ83767
	Primula malacoides virus	ABW82141
	Red clover cryptic virus 2	AGJ83765
	White clover cryptic virus 2	AGJ83763
**Collapsed group**		
	Heterobasidion partitivirus 3-ec1	ACO37245
	Heterobasidion partitivirus 12-an1	AHL25151
	Heterobasidion partitivirus 13-an1	AHL25153
	Heterobasidion partitivirus 15-pa1	AHL25162
	Helicobasidium mompa partitivirus V70	BAC23065
